# Multiple sclerosis, disease-modifying drugs and risk for adverse perinatal and pregnancy outcomes: Results from a population-based cohort study

**DOI:** 10.1177/13524585231161492

**Published:** 2023-04-18

**Authors:** Katharina Fink, Agnes Gorczyca, Peter Alping, Simon Englund, Susan Farmand, Annette M Langer-Gould, Fredrik Piehl, Kyla McKay, Thomas Frisell, Neda Razaz

**Affiliations:** Department of Clinical Neuroscience, Karolinska Institutet, Department of Neurology, Karolinska University Hospital, Stockholm, Sweden Centre for Neurology, Academic Specialist Centre, Stockholm, Sweden; Department of Clinical Neuroscience, Karolinska Institutet, Department of Neurology, Karolinska University Hospital, Stockholm, Sweden; Department of Clinical Neuroscience, Karolinska Institutet, Department of Neurology, Karolinska University Hospital, Stockholm, Sweden Department of Medicine, Solna, Clinical Epidemiology Division, Karolinska Institutet, Stockholm, Sweden Centre for Neurology, Academic Specialist Centre, Stockholm, Sweden; Department of Clinical Neuroscience, Karolinska Institutet, Department of Neurology, Karolinska University Hospital, Stockholm, Sweden; Center for Obstetrics and Pediatrics, Universitätsklinikum Hamburg-Eppendorf, Hamburg, Germany; Southern California Permanente Medical Group, Kaiser Permanente, Los Angeles, CA, USA; Department of Clinical Neuroscience, Karolinska Institutet, Department of Neurology, Karolinska University Hospital, Stockholm, Sweden; Department of Clinical Neuroscience, Karolinska Institutet, Department of Neurology, Karolinska University Hospital, Stockholm, Sweden; Department of Medicine, Solna, Clinical Epidemiology Division, Karolinska Institutet, Stockholm, Sweden; Department of Medicine, Solna, Clinical Epidemiology Division, Karolinska Institutet, Stockholm, Sweden

**Keywords:** Disease-modifying therapies, epidemiology, multiple sclerosis, pregnancy

## Abstract

**Background:**

There is a paucity of information on maternal multiple sclerosis (MS) and risk of adverse pregnancy and perinatal outcomes.

**Objective:**

The aim of this study was to determine the association between MS and risks of adverse pregnancy and perinatal outcomes in women with MS. In women with MS, the influence of exposure to disease-modifying therapy (DMT) was also investigated.

**Methods:**

Population-based retrospective cohort study on singleton births to mothers with MS and matched MS-free mothers from the general population in Sweden between 2006 and 2020. Women with MS were identified through Swedish health care registries, with MS onset before child’s birth.

**Results:**

Of 29,568 births included, 3418 births were to 2310 mothers with MS. Compared with MS-free controls, maternal MS was associated with increased risks of elective caesarean sections, instrumental delivery, maternal infection and antepartum haemorrhage/ placental abruption. Compared with offspring of MS-free women, neonates of mothers with MS were at increased risks of medically indicated preterm birth and being born small for gestational age. DMT exposure was not associated with increased risks of malformations.

**Conclusions:**

While maternal MS was associated with a small increased risk of few adverse pregnancy and neonatal outcomes, DMT exposure close to pregnancy was not associated with major adverse outcomes.

## Introduction

Multiple sclerosis (MS) is a chronic inflammatory disorder of the central nervous system affecting more women than men and typically manifests during reproductive ages.

Clinicians recommend discontinuation of disease-modifying therapies (DMTs) before conception unless the risk of disease worsening outweighs the risk to the foetus.^1^ Existing evidence suggests that women with active disease may continue treatments with first-generation, injectable DMTs, including interferon beta and glatiramer acetate until conception or even during pregnancy to control disease activity.^2,3^

Several studies have shown that MS appears to increase the risk of caesarean delivery,^4–7^ preterm birth,^6,8,9^ being born small for gestational age^4–6^ and foetal malformation.^8^ However, no increased risk of adverse pregnancy outcomes among women with MS has been reported.^7,10–13^ Only few studies have considered MS-specific factors (e.g. treatment, level of disability or relapse during pregnancy) and the possible influence on adverse outcomes.

Second-generation, so-called high-efficacy DMTs, including natalizumab, anti-CD20 antibodies and fingolimod, have been associated with several adverse pregnancy and neonatal outcomes.^14–16^ Specifically, fingolimod has been associated with an increased risk of foetal malformation,^14^ natalizumab with haematological abnormalities^16^ and anti-CD20 antibodies with severe maternal infections.^15^ In addition, infants exposed to DMT during pregnancy reported to have lower birth weight or length.^17,18^

Therefore, the objective of this study was to examine the associations between MS in pregnancy and risks of perinatal adverse outcomes in a nationwide cohort with population-based controls.

In women with MS, we also investigated whether in utero DMT exposure and MS-specific clinical factors were associated with increased risks.

## Methods

Using the person-unique national registration numbers of mothers and their offspring,^19^ individual information was obtained from the Swedish MS register, which covers approximately 80% of all MS patients in Sweden since 2000 and collects longitudinal data on treatment, disease course and disease activity;^20,21^ the Medical Birth Register (MBR);^22^ the National Patient Register^23^ and the Prescribed Drug Registry, which stores data on all drugs prescribed in ambulatory care and dispensed at a Swedish pharmacy since 1 July 2005.^24^ The Education Register and the Total Population Register were used to obtain information on maternal education and country of origin, respectively.^19,25^ The Swedish version of the International Classification of Diseases *10th Revision* (*ICD-10)* had been used to code diagnoses. The study was approved by the Regional Ethical Board of Stockholm, Sweden (No. 2017/700-31/4 and 2021-02384). As is common for pseudonymised linkages of national registers in Sweden, the need for individual informed consent was waived.

### Study participants

Women with MS disease onset before a live birth between 1 January 2006 and 12 December 2020 were identified through linkage of the Swedish MS Register, National Patient Register and the MBR. The reason for choosing 2006 as the start of the study was that information on Prescription Drug Register effective disease-modifying drugs became available in Sweden. Cases of MS derived from the Swedish MS registry were diagnosed by a neurologist. Furthermore, cases were identified from the National Patient Register using a validated algorithm, which has been shown to be highly sensitive.^26^ The ‘onset date’ for these patients was the date of first ICD code for MS. From Statistics Sweden, women free of MS were selected from the general population, using risk-set sampling. Women with and without MS could contribute multiple births to the analysis.

### Disease-modifying drug exposure

Using information from the Prescribed Drug Registry^24^ and the Swedish MS register, we were able to assess DMT exposure in women with MS. The exposed group consisted of births to women with MS exposed to interferon beta, fingolimod, natalizumab, glatiramer acetate and dimethyl fumarate within 3 months prior to conception and during pregnancy. Given the extended biological effects of rituximab, an exposure window of 6 months was defined for women exposed to rituximab prior to conception and during pregnancy. For natalizumab and rituximab, dates of infusion were collected from the Swedish MS registry. The unexposed group consisted of births to women with MS not exposed to any DMT within 12 months prior to pregnancy. The date of conception was obtained by subtracting the gestational age.

### Pregnancy and perinatal outcomes

Pregnancy outcomes were chosen a priori and included gestational diabetes, preeclampsia, maternal infection, antepartum haemorrhage/placental abruption, labour dystocia, obstetric anal sphincter injury, induction of labour, mode of delivery and postpartum haemorrhage (see Supplemental Table S1 for specific codes).

Perinatal outcomes included preterm birth (< 37 completed weeks’ gestation), spontaneous and medically indicated preterm birth, small for gestational age live births, low 5-minute Apgar score < 7, presence of major congenital malformation detected during the first year of life and the following neonatal complications (within 28 days after birth): infections, hypoglycaemia, jaundice and respiratory distress (see Supplemental Table S1 for specific codes).

Gestational age was estimated using the following hierarchy: ultrasonography offered no later than the early second trimester (89.8%); date of last menstrual period reported at the first antenatal visit (5.5%) and a postnatal assessment (4.8%). Medically indicated preterm was defined as being born preterm and having an induced onset of labour or a caesarean delivery before the onset of labour. Small for gestational age (SGA) live birth was defined as less than two standard deviations below the mean birth weight for gestational age, according to the Swedish ultrasound foetal growth chart.^27^ Induced abortion due to detected malformation is legal until 21 gestational weeks in Sweden and are therefore not included in the MBR.

### Other covariates

Maternal characteristics included age at delivery, region of origin, education level, cohabitation with a partner, parity, height, body mass index (BMI, kg/m^2^), smoking during pregnancy, year of delivery and maternal pre-existing chronic conditions, such as pregestational diabetes and hypertension. Parity was defined as the number of births by each mother (including the present birth). BMI was calculated using weight measured at the first antenatal care registration and self-reported height. BMI was categorized according to the World Health Organization as underweight (BMI < 18.5), normal weight (18.5–25), overweight (25–30), obesity grade I (30–35) and obesity grade II–III (⩾ 35.0).^28^ Information on cohabitation with the partner was obtained at the first antenatal visit. Mothers who reported daily smoking and snuff use at the first antenatal visit and at 30–32 gestational weeks were classified as smokers and snuff users. Information on induction of labour and mode of delivery was noted on standardized checkboxes at the onset of labour and after delivery, respectively.

### Statistics

We assessed the distribution of maternal characteristics of mothers with and without MS and those exposed to DMT and not exposed to DMT during pregnancy. Multivariable Poisson log-linear regression models were used to estimate adjusted risk ratios (RRs) and 95% confidence intervals (CIs). For the computation of 95% CI, generalized estimating equations, with an assumed independent correlation structure, were used to account for correlation between births from the same mother. Models were adjusted for maternal age, region of origin, education level, height, parity, smoking or snuff use during pregnancy and year of delivery. Pregnancy and neonatal outcomes in DMT exposed pregnancies were examined using multivariable Poisson log-linear regression models adjusting for maternal age, parity and year of delivery (**Supplemental analyses).**

The association between MS-related clinical factors on the pregnancy and perinatal outcomes was examined within the MS cohort with available information from the Swedish MS registry on disability, relapse during pregnancy and MS clinical course (relapsing–remitting, secondary progressive, primary progressive), using multivariable logistic regression. Disability was measured via the Expanded Disability Status Scale (EDSS) score assigned closest to the time of delivery (± 3 years). MS mothers were classified as having normal to mild disability (EDSS < 3), or moderate to severe disability (EDSS ⩾ 3). Relapses were recorded by the treating neurologist and defined as new or worsening of existing neurologic symptoms not associated with fever or infection that lasted for at least 24 hours and were accompanied by new neurologic signs. Finally, to examine if the relationship between maternal MS and early term/preterm delivery is due to elective caesarean sections, we excluded in a sensitivity analyses all elective caesarean sections at < 39 weeks gestation.

Data were analysed with the use of SAS software, version 9.4 (SAS Institute). No adjustments were made for multiple comparisons. Therefore, because of the potential for type I error, the results of this study should be considered exploratory.

## Results

The final sample included 29,568 pregnancies, of those 3418 pregnancies were to 2310 women with MS and 26,150 pregnancies to women without MS. A total of 1172 (34%) pregnancies with MS were exposed to DMT within 3 months (6 months for rituximab) prior to and during pregnancy. Of those exposed, 536 pregnancies were exposed to interferon beta, 138 to glatiramer acetate, 225 to rituximab, 302 to natalizumab and 35 to fingolimod (multiple exposure within 6 months prior to pregnancy is possible). Of all the pregnancies to MS mothers, 3057 also had available information on MS disease characteristics (Supplemental Tables S3). During pregnancy, 220 (7.2%) women experienced a relapse.

Compared with women without MS, women with MS were, on average, older at the time of delivery, nulliparous, born in the Nordic countries, smoked or were snuff users during pregnancy and had a higher education (Table 1). Among women with MS, those exposed to DMT within 3 months (6 months for rituximab) prior to and during pregnancy were more often nulliparous and born in the Nordic countries.

**Table 1.** Maternal and pregnancy outcomes according to maternal MS and maternal drug use during pregnancy in Sweden from 2006 to 2020.

Maternal and pregnancy characteristics	*N* (%)				
	Total (*N* = 29,568)	Without MS (*N* = 26,150)	With MS (*N* = 3418)	Not Exposed to DMT* (*N* = 2002)	Exposed to DMT (*N* = 1172)
Age (years)					
⩽19	80 (0.3)	73 (0.3)	7 (0.2)	6 (0.3)	1 (0.1)
20–24	1486 (5.0)	1316 (5.0)	170 (5.0)	101 (5.0)	60 (5.1)
25–29	7083 (24.0)	6280 (24.0)	803 (23.5)	461 (23.0)	288 (24.6)
30–34	11,604 (39.2)	10,195 (39.0)	1409 (41.2)	800 (40.0)	504 (43.0)
⩾35	9315 (31.5)	8286 (31.7)	1029 (30.1)	634 (31.7)	319 (27.2)
Country of birth					
Missing	32 (0.1)	28 (0.1)	4 (0.1)	3 (0.1)	1 (0.1)
Nordic	24,819 (83.9)	21,872 (83.6)	2947 (86.2)	1708 (85.3)	1024 (87.4)
Non-nordic	4717 (16.0)	4250 (16.3)	467 (13.7)	291 (14.5)	147 (12.5)
Education					
Missing	118 (0.4)	107 (0.4)	11 (0.3)	7 (0.3)	4 (0.3)
⩽9	1752 (5.9)	1560 (6.0)	192 (5.6)	122 (6.1)	66 (5.6)
10–11	1982 (6.7)	1771 (6.8)	211 (6.2)	139 (6.9)	55 (4.7)
12	7343 (24.8)	6558 (25.1)	785 (23.0)	439 (21.9)	286 (24.4)
13–14	4301 (14.5)	3787 (14.5)	514 (15.0)	304 (15.2)	166 (14.2)
⩾15	14,072 (47.6)	12,367 (47.3)	1705 (49.9)	991 (49.5)	595 (50.8)
Cohabitation with partner					
Missing	1414 (4.8)	1261 (4.8)	153 (4.5)	94 (4.7)	44 (3.8)
Yes	26,550 (89.8)	23,445 (89.7)	3105 (90.8)	1808 (90.3)	1078 (92.0)
No	1604 (5.4)	1444 (5.5)	160 (4.7)	100 (5.0)	50 (4.3)
Smoking					
Missing	961 (3.3)	849 (3.2)	112 (3.3)	67 (3.3)	33 (2.8)
No	27,127 (91.7)	24,016 (91.8)	3111 (91.0)	1818 (90.8)	1073 (91.6)
Yes	1480 (5.0)	1285 (4.9)	195 (5.7)	117 (5.8)	66 (5.6)
Snuff					
Missing	858 (2.9)	756 (2.9)	102 (3.0)	60 (3.0)	30 (2.6)
No	28,205 (95.4)	24,958 (95.4)	3247 (95.0)	1898 (94.8)	1120 (95.6)
Yes	505 (1.7)	436 (1.7)	69 (2.0)	44 (2.2)	22 (1.9)
Year of delivery					
2006–2009	6356 (21.5)	5512 (21.1)	844 (24.7)	602 (30.1)	155 (13.2)
2010–2013	7869 (26.6)	6899 (26.4)	970 (28.4)	537 (26.8)	357 (30.5)
2014–2017	8715 (29.5)	7751 (29.6)	964 (28.2)	513 (25.6)	386 (32.9)
2018–2020	6628 (22.4)	5988 (22.9)	640 (18.7)	350 (17.5)	274 (23.4)
Parity					
1	11,488 (38.9)	9986 (38.2)	1502 (43.9)	827 (41.3)	540 (46.1)
2	11,494 (38.9)	10,121 (38.7)	1373 (40.2)	820 (41.0)	470 (40.1)
3	4597 (15.5)	4188 (16.0)	409 (12.0)	263 (13.1)	125 (10.7)
⩾4	1989 (6.7)	1855 (7.1)	134 (3.9)	92 (4.6)	37 (3.2)
Height (cm)					
Missing	371 (1.3)	317 (1.2)	54 (1.6)	27 (1.3)	20 (1.7)
⩽159	3582 (12.1)	3254 (12.4)	328 (9.6)	181 (9.0)	124 (10.6)
160–164	7110 (24.0)	6331 (24.2)	779 (22.8)	453 (22.6)	269 (23.0)
165–169	8525 (28.8)	7515 (28.7)	1010 (29.5)	590 (29.5)	353 (30.1)
⩾170	9980 (33.8)	8733 (33.4)	1247 (36.5)	751 (37.5)	406 (34.6)
Pre-pregnancy BMI (kg/m^2^)					
Missing	1879 (6.4)	1669 (6.4)	210 (6.1)	126 (6.3)	66 (5.6)
<18.5	596 (2.0)	516 (2.0)	80 (2.3)	41 (2.0)	35 (3.0)
18.5–24.9	16,346 (55.3)	14,437 (55.2)	1909 (55.9)	1113 (55.6)	665 (56.7)
25.0–29.9	6918 (23.4)	6132 (23.4)	786 (23.0)	455 (22.7)	267 (22.8)
30.0–34.9	2632 (8.9)	2341 (9.0)	291 (8.5)	176 (8.8)	98 (8.4)
35.0–39.9	883 (3.0)	775 (3.0)	108 (3.2)	69 (3.4)	33 (2.8)
⩾40.0	314 (1.1)	280 (1.1)	34 (1.0)	22 (1.1)	8 (0.7)
Pregestational diabetes					
No	29,098 (98.4)	25,721 (98.4)	3377 (98.8)	1974 (98.6)	1162 (99.1)
Yes	470 (1.6)	429 (1.6)	41 (1.2)	28 (1.4)	10 (0.9)
Pregestational hypertension					
No	29,305 (99.1)	25,920 (99.1)	3385 (99.0)	1981 (99.0)	1161 (99.1)
Yes	263 (0.9)	230 (0.9)	33 (1.0)	21 (1.0)	11 (0.9)

MS: multiple sclerosis; DMT: disease-modifying therapy.

* Not receiving any disease-modifying drugs (DMTs) within 1 year prior to pregnancy.

Maternal MS was associated with higher risks of elective caesarean section, instrumental delivery, maternal infection and antepartum haemorrhage/ placental abruption, compared with pregnancies in women without MS (Table 2). After adjusting for potential confounder, neonates of women with MS had significantly higher risks of early term birth, medically indicated preterm birth (< 37 weeks) and being born small for gestational age compared with neonates of unaffected women (Table 2).

**Table 2.** Pregnancy and perinatal outcomes according to maternal MS in Sweden from 2006 to 2020.

Outcomes	*N* (%)			RR (95% CI)	
	Total (*N* = 29,568)	Without MS (*N* = 26,150)	With MS (*N* = 3418)	Crude	Adjusted^b^
Pregnancy outcomes					
Elective caesarean section	2841 (9.6)	2419 (9.3)	422 (12.3)	1.36 (1.22–1.51)	**1.37 (1.23–1.52)**
Emergency caesarean section	2426 (8.2)	2149 (8.2)	277 (8.1)	1.05 (0.93–1.19)	1.02 (0.91–1.15)
Instrumental delivery	1952 (6.6)	1660 (6.3)	292 (8.5)	1.40 (1.24–1.58)	**1.27 (1.13–1.42)**
Induction of labour	4828 (16.3)	4242 (16.2)	586 (17.1)	1.05 (0.97–1.14)	1.08 (0.99–1.17)
Gestational diabetes	470 (1.6)	429 (1.6)	41 (1.2)	0.77 (0.56–1.07)	0.90 (0.64–1.25)
Preeclampsia	783 (2.6)	701 (2.7)	82 (2.4)	0.90 (0.71–1.14)	0.89 (0.70–1.12)
Maternal infection	1370 (4.6)	1194 (4.6)	176 (5.1)	1.14 (0.97–1.35)	**1.20 (1.02–1.42)**
Antepartum haemorrhage/ placental abruption	249 (0.8)	209 (0.8)	40 (1.2)	1.45 (1.02–2.05)	**1.50 (1.05–2.14)**
Labour dystocia	3108 (10.5)	2755 (10.5)	353 (10.3)	0.98 (0.88–1.09)	0.91 (0.82–1.01)
Obstetric anal sphincter injury	741 (2.5)	668 (2.6)	73 (2.1)	0.84 (0.66–1.06)	0.73 (0.57–0.94)
Postpartum haemorrhage	2095 (7.1)	1840 (7.0)	255 (7.5)	1.06 (0.93–1.21)	1.03 (0.90–1.18)
Perinatal outcomes					
Early term (37–38 weeks)	5602 (18.9)	4853 (18.6)	749 (21.9)	1.19 (1.11–1.28)	**1.22 (1.13–1.31)**
Preterm birth (< 37 weeks)	1362 (4.6)	1171 (4.5)	191 (5.6)	1.24 (1.06–1.45)	**1.23 (1.04–1.45)**
Medically indicated preterm birth^a^	403 (1.4)	342 (1.3)	61 (1.8)	1.35 (1.01–1.81)	**1.41 (1.04–1.91)**
Spontaneous preterm birth^a^	953 (3.2)	823 (3.1)	130 (3.8)	1.20 (1.00–1.45)	1.17 (0.96–1.42)
Small for gestational age	1867 (6.3)	1613 (6.2)	254 (7.4)	1.21 (1.06–1.39)	**1.17 (1.02–1.34)**
Neonatal infection	358 (1.2)	311 (1.2)	47 (1.4)	1.16 (0.85–1.57)	1.18 (0.86–1.62)
Major malformation	1334 (4.5)	1175 (4.5)	159 (4.7)	1.03 (0.88–1.22)	1.07 (0.90–1.26)
5–minute Apgar score < 7	529 (1.8)	461 (1.8)	68 (2.0)	1.12 (0.87–1.45)	1.21 (0.93–1.57)
Neonatal hypoglycaemia	643 (2.2)	579 (2.2)	64 (1.9)	0.84 (0.64–1.09)	0.85 (0.65–1.11)
Neonatal jaundice	1312 (4.4)	1156 (4.4)	156 (4.6)	1.03 (0.88–1.22)	0.98 (0.82–1.16)
Neonatal respiratory distress	1056 (3.6)	925 (3.5)	131 (3.8)	1.08 (0.90–1.30)	1.06 (0.88–1.29)

MS: multiple sclerosis.

a We did not have data on spontaneous versus induced preterm birth in all preterm births, hence numbers do not add up.

b Adjusted for maternal age, country of origin, parity, education, smoking and snuff during pregnancy, year of birth and maternal height.

Statistically significant adjusted RR is given in bold.

Neonates of women exposed to DMT did not have a higher risk of congenital malformation compared with the nonexposed offspring (4.4% vs 4.7%; Table 3).

**Table 3.** Perinatal outcomes according to maternal drug use during pregnancy in Sweden from 2006 to 2020.

Outcomes	*N* (%)		RR (95% CI)	
	Not exposed to DMT* (*N* = 2002)	Exposed to DMT (*N* = 1172)	Crude	Adjusted^b^
Pregnancy outcomes				
Elective caesarean section	245 (12.2)	145 (12.4)	1.01 (0.86–1.20)	1.05 (0.88–1.24)
Emergency caesarean section	155 (7.7)	106 (9.0)	1.18 (0.94–1.47)	1.13 (0.90–1.42)
Instrumental delivery	156 (7.8)	104 (8.9)	1.16 (0.92–1.46)	1.13 (0.90–1.43)
Induction of labour	335 (16.7)	217 (18.5)	1.07 (0.92–1.26)	1.02 (0.87–1.20)
Gestational diabetes	28 (1.4)	10 (0.9)	0.61 (0.30–1.24)	0.54 (0.26–1.12)
Preeclampsia	45 (2.2)	29 (2.5)	1.08 (0.68–1.73)	1.04 (0.64–1.67)
Maternal infection	102 (5.1)	64 (5.5)	1.09 (0.80–1.49)	0.97 (0.71–1.33)
Antepartum haemorrhage/ placental abruption	23 (1.1)	12 (1.0)	0.93 (0.47–1.82)	0.96 (0.51–1.83)
Labour dystocia	189 (9.4)	129 (11.0)	1.17 (0.94–1.44)	1.09 (0.88–1.35)
Obstetric anal sphincter injury	51 (2.5)	18 (1.5)	0.60 (0.35–1.03)	0.58 (0.32–1.01)
Postpartum haemorrhage	141 (7.0)	96 (8.2)	1.15 (0.89–1.48)	1.13 (0.88–1.47)
Perinatal outcomes				
Early term (37–38 weeks)	442 (22.1)	256 (21.8)	0.99 (0.86–1.14)	1.02 (0.89–1.18)
Preterm birth (< 37 weeks)	103 (5.1)	74 (6.3)	1.20 (0.90–1.60)	1.20 (0.90–1.61)
Medically indicated preterm birth^a^	29 (1.4)	28 (2.4)	1.40 (0.84–2.33)	1.37 (0.83–2.27)
Spontaneous preterm birth^a^	74 (3.7)	46 (3.9)	1.07 (0.75–1.53)	1.09 (0.75–1.58)
Small for gestational age	149 (7.4)	82 (7.0)	0.91 (0.70–1.18)	0.92 (0.71–1.19)
Neonatal infection	22 (1.1)	19 (1.6)	1.48 (0.80–2.72)	1.49 (0.83–2.67)
Major malformation	95 (4.7)	52 (4.4)	0.93 (0.67–1.29)	0.88 (0.63–1.23)
5-minute Apgar score < 7	38 (1.9)	25 (2.1)	1.13 (0.68–1.87)	1.13 (0.67–1.93)
Neonatal hypoglycaemia	38 (1.9)	21 (1.8)	0.97 (0.57–1.67)	1.07 (0.63–1.81)
Neonatal jaundice	83 (4.1)	55 (4.7)	1.13 (0.81–1.58)	1.22 (0.87–1.71)
Neonatal respiratory distress	63 (3.1)	59 (5.0)	1.58 (1.12–2.24)	**1.52 (1.06–2.18)**

DMT: disease-modifying therapy.

a We did not have data on spontaneous versus induced preterm birth in all preterm births, hence numbers do not add up.

b Adjusted for maternal age, parity and year of birth.

* Not receiving any disease-modifying drugs (DMTs) within 1 year prior to pregnancy.

Statistically significant adjusted RR is given in bold.

Of the 52 cases of major congenital malformation identified in the DMT-exposed cohort, 34 cases were exposed to interferon beta/glatiramer acetate, 1 to fingolimod, 10 to rituximab and 7 to natalizumab. Offspring of women exposed to DMT had higher frequency of respiratory distress (5.0% vs 3.1%) compared with the nonexposed offspring. In the adjusted analyses, no statistically significant increased risk of adverse neonatal outcome was found between the two groups for any of the neonatal outcomes, except for a 52% higher risk of respiratory distress (aRR 1.52; 95% CI (1.06–2.18); Table 3).

Relapses during pregnancy were only associated with higher risks of medically indicated preterm births, but no increased risk of adverse neonatal outcomes were observed, compared with those without a relapse (Supplemental Table S2). Compared with women with normal/mild findings in neurological examination, women with moderate/severe impairment had a higher frequency of elective caesarean section (11.4% vs 17.8%), emergency caesarean section (7.3% vs 11.3%) and instrumental delivery (8.4% vs 10.7%; Supplemental Table S3). Compared with offspring to women with normal/mild findings in neurological examination, offspring to women with moderate/severe impairment had a higher risk for spontaneous preterm birth.

The frequency of emergency caesarean delivery, instrumental delivery and labour dystocia was higher among pregnancies to women with primary progressive MS compared with women with relapsing–remitting MS (Supplemental Table S4). However, due to small number of cases for each disease course, RRs could not be reliably calculated.

Supplemental Table S5 shows that after excluding elective caesarean section at < 39 weeks gestation, maternal MS is associated with higher risks of early-term delivery and SGA and a non-statistically significantly higher risk of preterm delivery.

## Discussion

In this nationwide cohort study, we observed higher risks of elective caesarean delivery, instrumental delivery, maternal infection and antepartum haemorrhage/ placental abruption in pregnancies with MS compared with non-MS controls. Furthermore, offspring of women with MS were at higher risks of early term birth, medically indicated preterm birth and being born small for gestational age. In contrast, exposure to DMT shortly before or during pregnancy was not associated with increased risks of pregnancy and perinatal complications, with neonatal respiratory distress being the only exception. Furthermore, we found no increased risk of congenital malformation among offspring of women with MS who were exposed to DMT compared with unexposed offspring born to mothers with MS.

Similar to our findings, higher odds of caesarean delivery in women with MS compared with women in the general population have been previously reported.^4–7^ It is not clear whether the decision for elective caesarean delivery is due to a definite medical indication or if the higher rate depends on obstetricians perceiving MS as an indication to early delivery. Furthermore, women with higher levels of disability (EDSS ⩾ 3) had an increased risk of elective caesarean delivery. This corroborates a previous, smaller study reporting a non-significant trend for an increased risk of a caesarean delivery with greater levels of disability.^13^ A contributing factor may be that neuromuscular weakness, physical MS-specific fatigue and spasticity might interfere with the labour process, prompting caesarean sections and instrumental delivery as seen in our study.

Previous studies have reported variable risk increases in different pregnancy complications among women with MS.^4–7,29,30^ We observed 1.2 times higher risk for maternal infections during pregnancy in women with MS compared with women without MS, which is in line with other studies^7–9^ and probably derives mainly from infections known to be associated to MS, for example, urinary tract infections.^7,12^ In our study, we further found an increased risk of antepartum haemorrhage and placental abruption, indicating a higher risk for defective placentation disorder in women with MS. This is a novel finding since earlier studies did not find evidence suggesting an association between MS and placental abruption.^7,10^ The underlying cause for this association is unknown.

We confirm previously reported associations between maternal MS and higher risk of preterm birth,^6,8,9^ and intrauterine growth restriction.^4–6,30^ However, there are also conflicting data on the association between maternal MS and newborn gestational age^4,7,10,12^ and intrauterine growth restriction.^7,9,12,13^ Nevertheless, in our study, even after excluding elective caesarean section at < 39 weeks, maternal MS was associated with early-term delivery and small for gestational age at birth. In line with other studies,^4,10,12^ we found no elevated risks of low 5-minute Apgar score, neonatal infections, respiratory distress and jaundice among offspring of women with MS. DMT before or during pregnancy did not display greater risks of adverse neonatal outcomes, with the exception of an increased risk of neonatal respiratory distress.

Finally, the risk for malformations did not seem to be increased when exposed to DMT within 3 (6) months before or during pregnancy. Most women were exposed to interferons and glatiramer acetate, followed by rituximab. As rituximab is currently the most prescribed DMT for MS in Sweden,^31^ which is unique in an international context, the data presented herein are of particular value. In total, 10 malformations were observed out of 225 pregnancies exposed to rituximab prior or during pregnancy, in line with the reported rates of congenital malformations in the general population according to EUROCAT.^32^ Future studies should examine in more detail the exposure to specific and newer DMTs and pregnancy and neonatal outcomes.

### Strengths and limitations

Our nationwide cohort study differs from previous studies examining the association of MS and its treatment on maternal and perinatal outcomes in a number of ways. First, in our study, we compared outcomes of women with MS on DMTs versus those with MS who were not exposed to DMTs, while previous studies compared DMT treatment in women with MS to a large reference cohort of women without MS. This approach reduces confounding by indication bias;^33^ however, it still remains a potential limitation of our results. Second, we were able to investigate if disability level and disease activity were associated with an increased risk for adverse pregnancy outcomes. Furthermore, relapse, as a proxy for possible steroid exposure during pregnancy did not increase the risk for adverse perinatal outcomes. Third, in this Swedish cohort, 19% of the exposed pregnancies were exposed to rituximab. Finally, we were able to adjust for a number of confounding variables not considered in previous studies, such as maternal pre-existing chronic conditions.

This study has several limitations. The Prescription Drug Register only provides information on drugs that have been dispensed from pharmacies, and information on compliance is not captured. However, infusions such as rituximab and natalizumab are given in clinic, with each infusion documented in the Swedish MS registry where data completeness for DMTs are described as high compared with medical chart review.^21^ We also lacked information about malformations subjected to induced abortions until gestational week 21, which may have influenced the estimated association between exposure to DMTs and risk of major malformation in the offspring towards the null. Finally, in our DMT exposed cohort, we cannot rule out the possibility of false negative results due to a lack of power to detect a meaningful difference. Despite the extensive amount of studies in this field, results are still conflicting and the impact of unmeasured confounders such as symptomatic treatments apart from DMTs needs to be assessed in future studies.

## Conclusion

In this study, we observed that maternal MS was associated with small increased risks of adverse delivery, pregnancy and neonatal outcomes, which may be due to disease-related factors, including comorbidities associated with MS and symptomatic treatments. However, in our study, we did not find evidence that exposure to DMT shortly before or during pregnancy is associated with increased risks of major adverse delivery and perinatal outcomes. Overall, the absolute risks for adverse outcomes were small and should not influence the MS patient’s family planning but raise awareness in health care providers monitoring pregnancies in women with MS.

## Supplemental Material

sj-docx-1-msj-10.1177_13524585231161492 – Supplemental material for Multiple sclerosis, disease-modifying drugs and risk for adverse perinatal and pregnancy outcomes: Results from a population-based cohort study Click here for additional data file. Supplemental material, sj-docx-1-msj-10.1177_13524585231161492 for Multiple sclerosis, disease-modifying drugs and risk for adverse perinatal and pregnancy outcomes: Results from a population-based cohort study by Katharina Fink, Agnes Gorczyca, Peter Alping, Simon Englund, Susan Farmand, Annette M Langer-Gould, Fredrik Piehl, Kyla McKay, Thomas Frisell and Neda Razaz in Multiple Sclerosis Journal
