# Supplementary material for: Multiple sclerosis, disease-modifying drugs and risk for adverse perinatal and pregnancy outcomes: Results from a population-based cohort study
Source: Mult Scler. 2023 Apr 18;29(6):731–40. doi: 10.1177/13524585231161492 (PMC10176621; doi:10.1177/13524585231161492)
Supplement: sj-docx-1-msj-10.1177_13524585231161492 – Supplemental material for Multiple sclerosis, disease-modifying drugs and risk for adverse perinatal and pregnancy outcomes: Results from a population-based cohort study [file sj-docx-1-msj-10.1177_13524585231161492.docx]

| **Table S1.** International Statistical Classification of Diseases and Related Health Problems, 10^th^ edition codes used to identify study exposure, outcomes, and covariates in the study. | |
| --- | --- |
| **Exposure** | **ICD codes** |
| Multiple Sclerosis | ICD-10: G35 |
| **Outcomes** | **ICD codes** |
| ***Pregnancy and delivery outcomes***^2^ | |
| Gestational diabetes | ICD-10: E10-E14, O24.0-O24.3 |
| Preeclampsia | ICD-10: O14-O15 |
| Maternal infection | ICD-10: O41.1, O98 |
| Antepartum haemorrhage | ICD-10: O44, O45, O46 |
| Postpartum haemorrhage | ICD-10: O67.0, O67.8, O72 |
| Labour dystocia | ICD-10: O62.0-O62.2, O62.8, O62.9, O66.9,  O63.1, O63.9 |
| Obstetric anal sphincter injury | ICD-10: O70.2, O70.3 |
| ***Neonatal outcomes***^1, 3^ | |
| Neonatal hypoglycaemia | ICD-10: P70.4 |
| Neonatal jaundice | ICD-10: P55-P59 |
| Neonatal respiratory distress | ICD-10: P22 |
| Neonatal infections | ICD-10: P35-P39 |
| Congenital malformation: |  |
| Minor malformations^4^ | ICD-10: Q17.0, Q17.5, Q18.0, Q18.1, Q25.0, Q27.0, Q31.5, Q32.0, Q52.3, Q53.0 to Q53.9, Q66.5 to Q66.9, Q69.0 to Q69.9, Q70.0 to Q70.9, Q76.0, Q79.9, Q82.5, and Q82.9 |
| Major malformations | ICD-10: Q00-Q99, except for the codes above for minor malformations |
| **Covariates** | **ICD codes** |
| Pre-pregnancy hypertension^5^ | ICD-10: I10 to I15, O10 and O11 |
| Pre-pregnancy diabetes | ICD-10: O24.4 |

^1^ Identified from the National Patient Register (Swedish cohort).

^2^ Identified from the Medical Birth Register and the National Patient Register.

^3^ Information on neonatal outcomes were restricted to the first 27 days of life.

^4^ Minor malformations as defined by the Swedish National Board of Health and Welfare.

^5^ Definition of pre-pregnancy hypertension also included self-reported information at the first antenatal visit.

| **Table S2. Perinatal outcomes according to relapse during pregnancy in Sweden from 2006 to 2020** | | | | | | |
| --- | --- | --- | --- | --- | --- | --- |
|  | **N (%)** | |  | **Risk Ratio (95 Confidence interval)** | |  |
| **Outcomes** | **No Relapse (n=2837)** | **Relapse (n=220)** |  | **Crude** | **Adjusted^b^** |  |
| **Pregnancy outcomes** |  |  |  |  |  |  |
| Elective caesarean section | 341 (12.0) | 30 (13.6) |  | 1.15 (0.79-1.67) | 1.39 (0.95-2.04) |  |
| Emergency caesarean section | 225 (7.9) | 12 (5.5) |  | 0.73 (0.41-1.31) | 0.82 (0.46-1.47) |  |
| Instrumental delivery | 242 (8.5) | 23 (10.5) |  | 1.24 (0.81-1.89) | 1.13 (0.72-1.78) |  |
| Induction of labour | 477 (16.8) | 52 (23.5) |  | 1.42 (1.07-1.89) | **1.39 (1.01-1.90)** |  |
| Gestational diabetes | 28 (1.0) | 2 (0.9) |  | - | - |  |
| Preeclampsia | 72 (2.5) | 0 (0.0) |  | - | - |  |
| Maternal infection | 144 (5.1) | 10 (4.5) |  | 0.90 (0.47-1.70) | 0.87 (0.43-1.79) |  |
| Antepartum hemorrhage/ placental abruption | 33 (1.2) | 3 (1.4) |  | - | - |  |
| Labour dystocia | 294 (10.4) | 22 (10.0) |  | 0.96 (0.63-1.49) | 0.94 (0.60-1.47) |  |
| Obstetric anal sphincter injury | 62 (2.2) | 4 (1.8) |  | - | - |  |
| Postpartum haemorrhage | 218 (7.7) | 12 (5.5) |  | 0.71 (0.40-1.27) | 0.75 (0.41-1.38) |  |
| **Perinatal outcomes** |  |  |  |  |  |  |
| Early term (37-38 wk) | 628 (22.1) | 48 (21.7) |  | 0.98 (0.73-1.32) | 1.08 (0.80-1.46) |  |
| Preterm birth (<37 wk) | 163 (5.7) | 13 (5.9) |  | 1.03 (0.58-1.81) | 0.94 (0.51-1.75) |  |
| Medically indicated preterm birth^a^ | 47 (1.7) | 9 (4.1) |  | 2.51 (1.23-5.12) | **2.48 (1.15-5.34)** |  |
| Spontaneous preterm birth^a^ | 116 (4.1) | 4 (1.8) |  | - | - |  |
| Small for gestational age | 210 (7.4) | 20 (9.1) |  | 1.23 (0.78-1.94) | 1.32 (0.83-2.10) |  |
| Neonatal infection | 42 (1.5) | 3 (1.4) |  | - | - |  |
| Major malformation | 138 (4.9) | 8 (3.6) |  | 0.77 (0.38-1.57) | 0.84 (0.41-1.73) |  |
| 5-min Apgar score < 7 | 59 (2.1) | 4 (1.8) |  | - | - |  |
| Neonatal hypoglycaemia | 49 (1.7) | 5 (2.3) |  | 1.32 (0.52-3.30) | 1.30 (0.51-3.29) |  |
| Neonatal jaundice | 132 (4.7) | 11 (5.0) |  | 1.07 (0.58-1.99) | 0.94 (0.48-1.86) |  |
| Neonatal respiratory distress | 111 (3.9) | 9 (4.1) |  | 0.92 (0.29-2.97) | 0.91 (0.28-2.97) |  |
| ^a^ We did not have data on spontaneous vs induced preterm birth in all preterm births, hence numbers do not add up.  ^b^Adjusted for maternal age, country of origin, education level, parity and year of birth. | | | | | | |

|  | | | | | | |
| --- | --- | --- | --- | --- | --- | --- |
| **Table S3. Perinatal outcomes according to baseline EDSS in Sweden from 2006 to 2020** | | | | | | |
|  | **N (%)** | |  | **Risk Ratio (95 Confidence interval)** | | |
| **Outcomes** | **EDSS <3 (N=2720)** | **EDSS ≥3 (N=337)** |  | **Crude** | **Adjusted^b^** | |
| **Pregnancy outcomes** |  |  |  |  |  | |
| Elective caesarean section | 311 (11.4) | 60 (17.8) |  | 1.67 (1.27-2.20) | **1.54 (1.15-2.06)** | |
| Emergency caesarean section | 199 (7.3) | 38 (11.3) |  | 1.72 (1.21-2.43) | **1.79 (1.26-2.55)** | |
| Instrumental delivery | 229 (8.4) | 36 (10.7) |  | 1.45 (1.02-2.05) | **1.42 (0.99-2.05)** | |
| Induction of labour | 469 (17.2) | 60 (17.8) |  | 1.03 (0.79-1.35) | 1.05 (0.80-1.39) | |
| Gestational diabetes | 26 (1.0) | 4 (1.2) |  | - | - | |
| Preeclampsia | 64 (2.4) | 8 (2.4) |  | 1.01 (0.48-2.10) | 1.19 (0.56-2.52) | |
| Maternal infection | 131 (4.8) | 23 (6.8) |  | 1.42 (0.91-2.21) | 1.35 (0.85-2.14) | |
| Antepartum hemorrhage/ placental abruption | 30 (1.1) | 6 (1.8) |  | 1.61 (0.67-3.88) | 1.53 (0.62-3.73) | |
| Labour dystocia | 288 (10.6) | 28 (8.3) |  | 0.78 (0.53-1.16) | 0.75 (0.50-1.13) | |
| Obstetric anal sphincter injury | 60 (2.2) | 6 (1.8) |  | 0.81 (0.35-1.87) | 0.85 (0.36-1.98) | |
| Postpartum haemorrhage | 204 (7.5) | 26 (7.7) |  | 1.03 (0.68-1.55) | 1.12 (0.74-1.69) | |
| **Perinatal outcomes** |  |  |  |  |  | |
| Early Term (37-38 wk) | 579 (21.3) | 97 (28.8) |  | 1.35 (1.09-1.68) | **1.26 (1.01-1.58)** | |
| Preterm birth (<37 wk) | 145 (5.3) | 31 (9.2) |  | 1.73 (1.17-2.54) | **1.80 (1.19-2.70)** | |
| Medically indicated preterm birth^a^ | 47 (1.7) | 9 (2.7) |  | 1.54 (0.75-3.14) | 1.64 (0.79-3.39) | |
| Spontaneous preterm birth^a^ | 98 (3.6) | 22 (6.5) |  | 1.80 (1.14-2.86) | **1.86 (1.14-3.05)** | |
| Small for gestational age | 204 (7.5) | 26 (7.7) |  | 1.03 (0.68-1.54) | 1.04 (0.69-1.57) | |
| Neonatal infection | 41 (1.5) | 4 (1.2) |  | - | - | |
| Major malformation | 134 (4.9) | 12 (3.6) |  | 0.72 (0.40-1.30) | 0.68 (0.37-1.27) | |
| 5-min Apgar score < 7 | 54 (2.0) | 9 (2.7) |  | 1.35 (0.66-2.72) | 1.11 (0.52-2.35) | |
| Neonatal hypoglycaemia | 45 (1.7) | 9 (2.7) |  | 1.61 (0.79-3.30) | 1.31 (0.61-2.81) | |
| Neonatal jaundice | 130 (4.8) | 13 (3.9) |  | 0.81 (0.46-1.43) | 0.76 (0.42-1.39) | |
| Neonatal respiratory distress | 100 (3.7) | 20 (5.9) |  | 1.61 (1.00-2.61) | 1.43 (0.86-2.39) | |
| ^a^ We did not have data on spontaneous vs induced preterm birth in all preterm births, hence numbers do not add up. | | | | | | |
| ^b^Adjusted for maternal age, country of origin, education level, parity and year of birth. | | | | | | |

| **Table S4. Perinatal outcomes according to MS type in Sweden 2006-2020** | | | | |
| --- | --- | --- | --- | --- |
|  | **N (%)** | | |  |
| **Perinatal outcomes** | **RRMS**  **(N=2,811)** | **SPMS (n=172)** | **PPMS (n=28)** |  |
| **Pregnancy outcomes** |  |  |  |  |
| Elective caesarean section | 336 (12.0) | 33 (19.2) | 3 (10.7) |  |
| Emergency caesarean section | 212 (7.5) | 15 (8.7) | 6 (21.4) |  |
| Instrumental delivery | 229 (8.1) | 26 (15.1) | 5 (17.9) |  |
| Induction of labour | 481 (17.1) | 29 (16.9) | 8 (28.6) |  |
| Gestational diabetes | 28 (1.0) | 2 (1.2) | 0 (0.0) |  |
| Preeclampsia | 65 (2.3) | 6 (3.5) | 1 (3.6) |  |
| Maternal infection | 147 (5.2) | 4 (2.3) | 0 (0.0) |  |
| Antepartum haemorrhage/ placental abruption | 32 (1.1) | 1 (0.6) | 1 (3.6) |  |
| Labor dystocia | 289 (10.3) | 19 (11.0) | 4 (14.3) |  |
| Obstetric anal sphincter injury | 58 (2.1) | 6 (3.5) | 1 (3.6) |  |
| Postpartum haemorrhage | 209 (7.4) | 15 (8.7) | 1 (3.6) |  |
| **Perinatal outcomes** |  |  |  |  |
| Early Term (37-38 wk) | 619 (22.0) | 45 (26.2) | 7 (25.0) |  |
| Preterm birth (<37 wk) | 162 (5.8) | 11 (6.4) | 2 (7.1) |  |
| Medically indicated preterm birth^a^ | 49 (1.7) | 5 (2.9) | 1 (3.6) |  |
| Spontaneous preterm birth^a^ | 113 (4.0) | 6 (3.5) | 1 (3.6) |  |
| Small for gestational age | 204 (7.3) | 16 (9.3) | 6 (21.4) |  |
| Neonatal infection | 45 (1.6) | 0 (0.0) | 0 (0.0) |  |
| Major malformation | 136 (4.8) | 8 (4.7) | 1 (3.6) |  |
| 5-min Apgar score < 7 | 57 (2.0) | 3 (1.7) | 2 (7.1) |  |
| Neonatal hypoglycaemia | 47 (1.7) | 4 (2.3) | 1 (3.6) |  |
| Neonatal jaundice | 130 (4.6) | 11 (6.4) | 1 (3.6) |  |
| Neonatal respiratory distress | 108 (3.8) | 11 (6.4) | 1 (3.6) |  |

| **Table S5. Perinatal outcomes according to maternal multiple sclerosis, excluding those with elective caesarean section at <39 weeks gestation in Sweden from 2006 to 2020** | | |
| --- | --- | --- |
|  | **Risk Ratio (95% Confidence interval)** | |
| **Outcomes** | **Crude** | **Adjusted^b^** |
| **Perinatal outcomes** |  |  |
| Early term (37-38 wk) | 1.14 (1.05-1.24) | 1.18 (1.08-1.29) |
| Preterm birth (<37 wk) | 1.19 (1.00-1.43) | 1.16 (0.96-1.40) |
| Small for gestational age | 1.22 (1.06-1.41) | 1.19 (1.03-1.37) |
| ^a^ We did not have data on spontaneous vs induced preterm birth in all preterm births, hence numbers do not add up. | |  |
| ^b^Adjusted for maternal age, country of origin, parity, education, smoking and snuff during pregnancy, year of birth and maternal height. | | |
